# Cultural factors influencing COVID-19-related perceptions and behavior, seen from immigrants’ own perspective – a qualitative study in Norway

**DOI:** 10.1186/s13690-024-01327-z

**Published:** 2024-07-19

**Authors:** Solveig Vederhus, Eirik Myhre, Esperanza Diaz, Liv Grimstvedt Kvalvik

**Affiliations:** Department of Global Public Health and Primary Care, University of Bergen, Bergen, Norway

**Keywords:** Health behaviour, Culture, Cultural competence, COVID-19, Immigrant

## Abstract

**Background:**

Cultural factors are often mentioned as a possible explanation for the observed differences between immigrant populations compared to general populations with regards to COVID-19 disease burden and vaccination rates, but usually without any further exploration of what this entails. This paper aims to capture the thoughts of immigrants themselves and explore how they think culture may or may not have affected vaccination rates and health behavior during the pandemic.

**Methods:**

We performed qualitative interviews with 18 immigrants from Poland, Somalia and Sri Lanka living in Norway. Group interviews and individual interviews were transcribed and analyzed using systematic text condensation.

**Results:**

We identified four main themes the participants thought could influence spread of infection and vaccine hesitancy: cultural factors, transcultural factors, host society factors, and other personal factors. Social habits, religious traditions, attitudes towards and trust in the healthcare system, sense of community and societal duty were understood as cultural factors that influenced health behavior and vaccination hesitancy. However, different cultural factors could have varied impact on immigrants’ behavior related to COVID-19 and possibly other health settings for different immigrant groups. In addition, we found examples of other factors related to being ‘between cultures’, and we found structural and socioeconomic factors not linked to culture.

**Conclusions:**

Our paper brings awareness to how rules and guidelines may hit harder and interfere more in the way of life in some communities than others. In the continued work towards equity in health promotion and healthcare services, policymakers ought to keep the existence of such cultural differences in mind, to be able to make policies well fitted to ensure good health and quality of life for all.

**Supplementary Information:**

The online version contains supplementary material available at 10.1186/s13690-024-01327-z.

**Table:** 

**Text box 1. Contributions to the literature**
• Norwegian pandemic regulations affected the ways of life of some immigrant groups more than others.
• Examples of different cultural factors existing for different immigrant groups, that affect their COVID-19-related behavior.
• Knowledge about this is needed in order to further understand the effects of cultural differences on health-related behavior, and to ensure equal access to health care services for all groups of society.

## Background

Immigrants have been disproportionately impacted by COVID-19, in terms of cases, hospitalizations and deaths in several European countries [1], as well as being subject to inequities in delivery and uptake of vaccines [2]. In Norway, three times as many cases of COVID-19 infection were confirmed among people with immigrant backgrounds, compared to people born in Norway, and the highest rates of infections were registered among immigrants from Pakistan, Somalia, and Iraq [3]. There was also a large variation in vaccination rates among different immigrant groups. For people born in Poland, Romania and Lithuania COVID-19 vaccination rates were, by October 2021, approximately 45%, compared to 92% among those born in Sri Lanka and Vietnam. In comparison, vaccination coverage among people born in Norway with Norwegian parents was approximately 93% [4].

Connections between immigrant background and disease are complex, and not identical for all immigrant groups. Socioeconomic factors like family situation, educational level, housing, and income may impact immigrant groups differently – depending on socioeconomic level, degree of integration, knowledge of language, national and local policies and trust in the system [5].

Lack of information has been proposed as an explanation for differences in disease prevalence among immigrant groups [6, 7]. Information campaigns in several languages were initiated early in the pandemic, and several voluntary organizations actively reached out to specific immigrant groups [8]. However, the overrepresentation of immigrants in COVID-19 cases and hospitalizations was stable throughout the pandemic in Norway [3]. Furthermore, despite targeted campaigns, many immigrants did not feel the measures taken by the government addressed their everyday life challenges [9].

Culture includes social behavior, norms, knowledge, attitudes, art, rules, traditions and customs within one group [10]. Several studies have pointed at cultural factors as a possible explanation for the partially unexplained gap between burden of disease among immigrants and non-immigrants, without further exploring what lies in the term culture in this context [6, 11].

The Norwegian government used the term “collective responsibility” as incentive to follow guidelines during the pandemic [12]. The concept of cultural tightness is used to describe differences in strength of social norms and degree of tolerance of deviance from them [13]. According to previous literature, factors linked to culture like level of trust, change over time [14].

A previous study tried to give some ideas of what culture might be, without actually looking into it specifically [7]. In a study in Oslo, the authors suggest that cultural factors, “for example other norms of physical and social contact, or systematic misunderstandings regarding infection control” might in itself be a factor for the spread of COVID-19 [15]. A Norwegian report could not explain the differences in attitudes towards COVID-19 vaccines between immigrants and the general population as a whole by factors like education, trust, length of residence or mental health alone, and concluded that there was a need for qualitative studies investigating what lies behind these differences [16]. Another Norwegian study looking at vaccination among different immigrant groups also pointed out this complex interconnection between socioeconomic factors, country of origin and other factors that could influence willingness to get vaccinated [4]. In summary, culture is often referred to as an important part of the explanation of the high burden of disease for some immigrant groups, but studies do not clarify what is meant by “culture”. Furthermore, no study has specifically asked immigrants themselves what they consider to be cultural explanations related to the extra burden during the pandemic. To close this gap, this study aims to achieve new knowledge of immigrants’ own understanding of possible causes for a higher disease burden and lower vaccination coverage among them, with a special interest in understanding what immigrants themselves consider ‘cultural factors’ as opposed to other personal and system-related factors in the host society. Such knowledge will be useful in the case of future pandemics and could help create a more equitable healthcare system.

## Method

In this qualitative study we explored the beliefs and experiences of three different immigrant groups, regarding culture as a factor in the spread of COVID-19 and vaccine hesitancy. A qualitative design enabled us to understand immigrants’ own perspectives, and more specifically what they labeled as cultural factors as opposed to other factors. In this study we use the term culture broadly, in relation to ways of thinking, communicating and behaving [10].

We conducted group interviews and individual interviews with a total of 18 participants. By immigrant in this study, we mean a person born in another country, with parents also born abroad. Statistics Norway uses this definintion [17]. Immigrants from three countries – Poland, Sri Lanka and Somalia were chosen because they represent some of the larger immigrant populations in Norway [18], and because they all had different COVID 19-related statistics. For Poland, it was a relatively low rate of registered vaccinations, while for Sri Lanka it was a relatively high rate of vaccination [4]. Somalia had higher rates of confirmed cases and hospital admissions compared to the rest of the population [3]. The interview guide (presented in supplementary material) was adapted to each nationality to present the statistic of that immigrant group.

Establishing contact with possible participants was challenging. Thus, several recruitment forms were used. A group of five Somali women was recruited through established contacts at the Pandemic Centre at the University of Bergen (UiB). Further participants were recruited through local organizations, social media and direct contact by email. A group interview was conducted with five Sri Lankan immigrants. Three individual interviews were conducted with Polish immigrants before a group interview with four participants was performed. A Somali male was also recruited for an individual interview.

In total, six Somali, five Sri Lankans and seven Poles were interviewed, for a total of 18 participants divided between seven interviews. Details about the participants are shown in Table 1.

**Table 1:** Participant statistics by country of origin, August 2022 to February 2023, Bergen, Norway

Country of origin	Poland	Somalia	Sri Lanka
Gender distribution	5 women, 2 men	5 women, 1 man	1 woman, 4 men
Age in years, range (average)	40–72 (52)	22–53 (39)	40–58 (52)
Years living in Norway, range (average)	5–40 (16)	11–20 (16)	23–38 (33)
Profession groups	1 engineer, 1 retiree, 2 construction workers, 1 unemployed, 2 self-employed	1 healthcare worker, 1 on leave, 3 in education, 1 student with part time-job	1 IT worker, 1 technical supervisor, 1 technician, 1 self-employed, 1 data analyst

Demographics and reasons for migration varied among the participants recruited from each of the chosen groups. Though we aspired to recruit as demographically diverse participants as possible, it proved challenging. We prioritized getting at least one informant of each gender from each nationality.

We conducted semi-structured interviews at locations preferred by the participants, with one or both first authors present, depending on localization and accessibility. Geographical and chronological constraints both for authors and participants limited us from performing all interviews with both first authors present. When both interviewers were present, they were both active during the session. Both first authors were present for the Somali group interview and for two Polish individual interviews. Only one of the first authors was present for one individual Polish interview, one Somali individual interview and for the Sri Lanken and Polish group interviews. Participants chose their preferred language for the interviews. A professional interpreter was used during the Somali group interview, while in the Polish group interview one participant translated for others when needed. The rest of the interviews were conducted in Norwegian. Interviews lasted between 30 and 90 min, were audio recorded, and stored and transcribed in the University of Bergen’s safe data storage space.

Systematic text condensation (STC), according to Malterud [19] was used to analyze the data, using an inductive approach for analyses. Transcripts were read and re-read to get an overview of the material.

Units of meaning were identified and organized into codes such as “social culture”, “uncertainty around vaccination”, or “language barriers”. The first two authors went through all codes and identified subthemes and themes. These were discussed with the other authors, finally ending up with identifying four main themes; ‘cultural factors’, ‘transcultural factors’, ‘host society factors’, and ‘other personal factors’.

Given the size of our sample, the subthemes and themes were largely common to all migrants, but the content of some themes and subthemes was explained differently for some groups. In those cases, we tried to identify specific trends and present them as group statements under the given theme/subtheme to exemplify heterogeneity within the migrant community.

## Results

We identified four main themes the participants thought could influence spread of infection and vaccine hesitancy. All three groups mentioned several *‘cultural factors’* they believed could influence disease burden and vaccination rates. They also mentioned *‘transcultural factors*’ related to being influenced not only by the situation in Norway, but also in their country of origin. In addition, the immigrants mentioned factors that they did not connect to culture, these were ‘*host society factors’*, and *‘other personal factors’.*

### Cultural factors

When asked about cultural factors all groups stated that social habits, religion, and sense of community within the groups could influence the spread of COVID-19 and willingness to get vaccinated. The Sri Lankan and the Somali groups also talked about societal duty or responsibility, and attitudes towards the Norwegian healthcare system as part of their culture. These sub-themes are all related to behavior, norms, attitudes, rules and traditions within the groups.

#### Hospitality and social habits in the daily life

All three groups spoke about their social habits, but the ways of being social varied among them. The Somali group described themselves as more social and hospitable than Norwegians. *“We are hospitable people. It is common to visit each other at home in large groups. Norwegians often have only one family over, but us Somalis usually have many families visiting at once” (male, Somalia). “We might be more social and visit each other more. We like to come together. Many men like to meet up in cafes and talk” (female, Somalia).* It was also mentioned that during the pandemic many struggled with loneliness as common meeting places for Somalis closed. They also said that it took some time to understand that their social habits could lead to the spread of the virus. *“It was only after a while that we understood that visiting each other could lead to more people getting infected” (female, Somalia).*

Social gatherings were presented as important for the Sri Lankans as well. However, during the pandemic, they quickly adapted, and stopped all social gatherings. *“In our culture, we have a lot of different social gatherings and a lot of that was shut down during the pandemic” (male, Sri Lanka). “Personally, I have stopped visiting people. I feel like I need to take back that culture again*” *(female, Sri Lanka).*

One Polish participant pointed out that it was important to come together with family and friends. *“I think many people just said, ‘I don’t care, just come, we need to celebrate together’” (female, Poland).* The Polish group also described different attitudes towards social distancing “*We didn’t like it when we couldn’t come together. Here in Norway, you like to keep your distance. We like to kiss each other, and hug” (female, Poland).*

#### Religious traditions, rituals, and gatherings

Both in the Polish and the Somali groups the participants viewed themselves as more religious than Norwegians, and this religiosity was understood as part of their culture. *“For the Somali people, 99,9% are Muslim, so the culture is Islam, if you know what I mean. It is what we have been taught, and what is good custom, in the eyes of God” (male, Somalia).* Religious traditions and important rituals were not always compatible with COVID-19 guidelines. *“When a person with Islamic background dies, the person is brought to the mosque, and we have to be there and pray” (female, Somalia).*

One Polish participant talked about the church as an institution, with influence on its members. “*In a Catholic country like Poland, what the church and the bishop say and allow also means something. It could also influence the people who have moved to Norway. The Polish priests here are loyal to their bishops back in Poland” (female, Poland).* Another participant mentioned that there could be some superstition within the church. *“Some bishops meant that the holy spirit protects us from the virus” (female, Poland)* However, this was not the case for all Polish participants - one participant also mentioned that the church in Poland is varied and divided, and that not all Poles view themselves as religious.

#### Sense of community within the groups

All groups talked about relations with others from the same country, either as family or as a community from a given country. The Somali group described a sense of community among Somalis in Bergen, a strong feeling of “duty” toward each other. *“It is in our culture, that when someone is in need, you have to help that person, no matter what” (male, Somalia).* They felt the need to help and protect not only friends and family, but strangers as well. *“My sister heard about a Somali man living alone. We didn’t really know him, but we heard that he had fallen sick, and couldn’t cook, so we brought him food” (male, Somalia)*. The Somalis also had an extended definition of family – friends and extended relatives were also included. *“If one gets infected the whole family gets infected. And ‘the whole family’ is not only four but can often be more than 20 people” (female, Somalia).*

The participants also described that in Somali culture visiting when someone is sick is an important duty. “*Yes, you have to visit when someone is sick. It is very important. That person should not be alone. You feel guilty if you do not visit them” (female, Somalia).* This could interfere with adherence to rules and regulations. *“When someone was discharged from the hospital, we would visit that person at home, and several would visit. One did not really think ‘only one or two can visit’* ” *(female, Somalia).*

The Sri Lankans described a ‘looser’ connection among themselves, although many appreciated a sense of community within the Sri-Lankan school. They described a need to come together to speak their own language and teach their children traditions from their home country. *“I think we are very lucky to be part of a nice group. We get a lot of support from each other here too” (male, Sri Lanka).*

Informants from Poland described a more individualistic social culture, where the close family is most important. However, they also talked about a connection, somehow looser, with other Polish immigrants. One participant mentioned the Polish church as a meeting place. *“The church was a good place to start. Getting to know someone who speaks the same language, and who understands my situation” (female, Poland)*.

#### Societal duty and responsibility

Societal duty and responsibility were understood as a part of the culture among Sri Lankans. They described shared responsibility towards others as a motivation to get vaccinated. *“It is for your own safety and other people’s safety. When you haven’t gotten the vaccine, you could spread the virus to others” (female, Sri Lanka).* One participant mentioned that if you are a part of the Norwegian society, you have a duty to follow the rules and regulations in that society. *“Following the government’s rules, whether we are in Norway or another country, is part of our culture. Wherever we are” (male, Sri Lanka).* “*In our culture – one listens to the authorities.” (female, Sri Lanka).*

In general, the Sri Lankans viewed it as important to be a ‘good, respectable citizen’, wherever one resides. *“Our group from Sri Lanka is more motivated than groups from other countries to be a part of society as quickly as possible, I’ve seen some of that. We adopt the culture of Norwegians automatically*” *(female, Sri Lanka).* They explained that this is how they are raised from childhood – that you must contribute to society in any way you can. They also adhere strictly to rules, and ‘don’t want to be caught in front of others’, connecting it to perceptions of respectability.

In the Polish group, it was mentioned that Poles in Norway might not share this responsibility towards society as part of their culture. “*How you are engaged in society is totally different. You are more on your own. Family maybe, but not further out than that” (female, Poland). “Societal consequences, ‘I’m not doing this for myself, but for all, in solidarity’. I think many Poles lack this kind of understanding” (female, Poland).*

One participant explained that some might not feel the need to take the vaccine. *“Some would say* ‘*We are only our family – we isolate ourselves, and it will all be okay’” (female, Poland).* This was linked to a historical lack of trust in the authorities. The participants explained that during the communist era, they could not rely on the government or the state for help, so they had to rely on themselves. This, they believed, has influenced how many Poles view the world today. They mentioned voluntary community work (“dugnad”) in Norway as something that was unfamiliar to them. *“Another thing is voluntary work – that is a huge cultural difference. In the communist era, voluntary work became something we ‘had to do’, something that was demanded of us. That could explain cultural differences here. We always solve our problems on our own” (male, Poland).*

#### Use of traditional medicine

Both the Somali and the Sri Lankan groups mentioned that traditional medicine was used during the pandemic. *“From the culture we have medicine and our own recipes for things that are good for the body, and things to help if you are sick or have a fever” (male, Somalia).* The Sri Lankan participants said that they try herbal remedies for illness or fever, before consulting a doctor. “*We try treating with natural medicine first. In our culture we use natural medicine before we use biological medicine” (Female, Sri Lanka). “The same goes for a sprained ankle. We do ‘home medicine’ with exercises and massages and such, before we consult a surgeon*” *(male, Sri Lanka).* Neither group indicated that these ‘primary measures’ would stop them from reaching out to public healthcare when necessary.

#### Attitudes towards the Norwegian healthcare system

Culture is also linked to expectations in health-care provision. Both Sri Lankan and Polish participants mentioned being used to a more active approach to i.e., antibiotics and surgical procedures than the more conservative, “hands-off” approach in Norwegian public healthcare.

The Polish participants considered this an important reason for why many avoid or do not trust Norwegian healthcare and prefer consulting private healthcare in Poland. One participant mentioned that *“we do not generally trust the healthcare system here. It is a cultural difference, that we are skeptical of the Norwegian healthcare system” (female, Poland)*, while another argued that trust or lack of trust is related to how long one has lived in Norway. *“One shares the same trust that Norwegians have, when one has lived here for a long time” (female, Poland).*

Many Polish participants talked about making health choices independently. *“Many Poles bring medication and antibiotics from Poland and keep it here for future needs, and think they know better (female, Poland). “Many consult google instead of going to the doctor” (female, Poland).*

The Polish participants thought Norwegian health-care workers have a different way of communicating “*We want more direct and technical communication. It is hard to understand nuances” (female, Poland).* This could sometimes lead to misunderstandings. They also mentioned different expectations in meeting with healthcare providers. *“Partial sick note! It’s unheard of. What do they mean by that? Even though I have sick note I must go to work?” (female, Poland).* When the Polish group was asked about measures to increase vaccination, several participants believed the government should have made it mandatory and said ‘you have to’ instead of ‘you ought to’.

The Sri Lankans described following advice from healthcare providers as part of their culture. *“The people I know are very good at following such advice from their doctors and physiotherapists and doing what they should” (female, Sri Lanka).* They also spoke of a generally good impression of and trust in the Norwegian healthcare system.

### Transcultural factors

All three groups were also influenced by the society they left behind in their country of origin, and this was especially clear during a pandemic when different countries had different situations and recommendations. Participants described factors from their own culture, how they viewed the Norwegian culture – and a feeling of being “between cultures”. This aspect was defined as “transcultural factors”.

#### Making sense of COVID-19 information from two countries

Several participants mentioned receiving information about the pandemic both from their country of origin and from Norwegian sources. It could be through news, family and friends. This could sometimes cause confusion. *“In Poland, the politicians would recommend the vaccine, although they had not taken it themselves. People were skeptical. I think the skepticism was higher in Poland than in Norway” (female, Poland).* Among Poles there was an impression that many Poles live a parallelized life in Norway, with perhaps a stronger connection to Poland than to their surroundings in Norway. One participant pointed out that even well integrated immigrants might find it easier to follow the news from home. “*I know people who are very well integrated, with children in the Norwegian school, and a job and stuff. They still know better what goes on in Poland, both politically and in general” (female, Poland).*

The Sri Lankan group followed news both from their home country and from Norway, but they did not report the same confusion. “*My mother-in-law called me and said she heard that 26 people died of the vaccine in Norway. There were rumors going around globally. No, it’s not true. Very few people died in Norway – but the rumors are still going around you know?” (female, Sri Lanka*).

The Somali participants mentioned that information from the Norwegian government would be spread by word of mouth, which could lead to misinformation circulating. *“There are a lot of ‘words on the street’. It’s always like, one person hears it from another person, that corona is this and that, and that you might need to do this and that. You know, rumors on the street. People might not have read at all, only heard about covid from others, and then information has circulated” (male, Somalia).* One participant mentioned that although he had frequent contact with family back in Somalia, they were impacted by the pandemic later than Norway. *“At first, it started in Europe, and then it took a long while before I heard about covid in my home country. ‘Yes, now it has reached us as well’. Because I still keep in touch with the people down there you know” (male, Somalia).*

#### Mistrust and conspiracy originating from the home country

Trust seemed to migrate with individuals from one country to another. In the Polish group, many participants talked about conspiracy theories being spread, and that some did not trust official information coming from the Norwegian government during the pandemic. *“Polish people in general do not trust authorities*” *(female, Poland*). This was also put into context of the communist era. One participant mentioned that the general level of people who are against vaccines might be higher in Poland, and circulation of rumors that vaccines give autism and other side effects. *“I think they believe they should not trust anyone, and that they know better. Many are used to not trusting Norwegians” (female, Poland).* However, some of the Polish participants did not share the impression that the general mistrust in the vaccine was higher among Polish immigrants.

In contrast to this, Sri Lankan participants mentioned trusting the Norwegian government and health institutions during the pandemic and adhering to guidelines. *“We follow the government’s rules. When a message pops up saying, ‘it’s time to book an appointment for your second vaccine dose’, then we book an appointment, right? We are used to vaccines” (female, Sri Lanka).*

#### Integration level

The participants experienced different levels of connection to Norwegian society. This was related to degree of connection to society in general, and to language proficiency on the individual basis. The Sri Lankan participants felt that in general Sri Lankans in Norway had a good understanding of Norwegian language. Among both the Somali and Polish participants there were more impressions of people in their groups struggling with substantial language barriers. *“For some it is hard to understand the language in the news” (male, Somalia)*. *“Many cannot communicate well in Norwegian” (female, Poland)*.

One Polish participant pointed out that families with children in school might have more contact with Norwegians and the Norwegian system, but Polish people living alone might only interact with other Poles – at work, in church and in their free time. They described a pattern of many Poles maintaining a strong connection to their home country while interacting minimally with Norwegian society. *“Very many Poles live kind of both places. When they come here now [after the 2004 European Union (EU) changes] they often only speak Polish at work. They practically just commute from Poland” (female, Poland). “They talk about ‘going back home’ for maybe 10, 12 years, and they are still here” (female, Poland).*

The Sri Lankans felt their group was well integrated into Norwegian society. *“Most of us are established, have gone to school and have higher education and jobs and such” (male, Sri Lanka).* They also pointed to the importance of the next generation; *“our children, who are grown up now, are mixing with the majority population of Norway and follow their system. They will keep up with things and tell us ‘You need this and that’” (male, Sri Lanka).* They also pointed out societal responsibility preceding successful integration *“They have to allow us to come also. You cannot just say ‘they are not integrated, they are not integrated’, you have to allow – and say welcome. If you don’t do that, integration doesn’t work” (male, Sri Lanka).*

### Host society factors

The participants mentioned factors that they did not directly relate to culture or their country of origin. The common link between these was that they were all related to navigating the system in Norway as host society – and were therefore grouped as “host society factors”.

#### Access to language courses

In addition to language proficiency being a personal factor closely linked to integration, lack of access to language courses was mentioned as a factor that negatively impacted their pandemic response. After Poland became a part of the EU in 2004, the Polish came as labor immigrants, and were not offered a free language course. For most work immigrants these days there are not enough apparent advantages to taking the language courses considering the price. *“The language course itself was free until 2004. Back then there were more who went through it” (female, Poland). “But when she needed to speak Norwegian to deal with Norwegian customers to her business, she had an incentive to learn the language*” *(female, Poland).* On the opposite side, the Sri Lankans saw it as very useful that they got free access to language courses when they arrived in Norway to learn the local language. *“I think it was a good thing that we had to learn Norwegian when we arrived” (male, Sri Lanka).*

Language was also linked to health literacy, which they meant was not taken into account when the authorities gave information. Several Somali participants mentioned that there could be misunderstandings and confusion, particularly early on. *“The symptoms were difficult to understand in the beginning, and people said many different things” (female, Somalia). “There was a lot of confusion. Some would for example wear the facemask wrong without knowing it” (male, Somalia).*

#### Appropriateness of information about COVID-19

Appropriateness relates to how well the information is catered to its recipients in the different country backgrounds, both in relation to language/translation, and the means by which it is transmitted. Several informants reported barriers in relation to the information about COVID-19 given in Norway, often linked to language proficiency. However, the barriers were also linked to the appropriateness of the information. Somali participants emphasized challenges with obtaining quality information regarding COVID-19, and the positive impact of translating and distributing of official guidelines in the Somali community by Somali immigrants in social media groups. “*We didn’t find concrete information in our language. That could be the cause” (female, Somalia). “You got more information on Facebook about rules, and you knew what would happen tomorrow. So, it became easier after a while (female, Somalia).*

A Polish participant who knew Norwegian well pointed out that it was not always easy to find concrete information in Polish and felt the information in Norwegian was more accurate. *“When you wanted to check the requirements for travel, it was safer to check the information in Norwegian. Because I understand Norwegian, and the information was more accurate. I tried finding the original information because it was often more precise and longer. Then I might use the Polish version as a supplement. It was also not always easy to find the Polish version” (female, Poland).*

Although most Sri Lankans had good fluency in Norwegian and found it easy to obtain and understand COVID19-information, there were also some misunderstandings regarding side effects of the vaccines in the Sri Lankan environment. One participant mentioned that some might not have gotten vaccinated because they had an underlying condition, like diabetes, fearing the vaccine would make the underlying condition worse.

#### Discrimination

The Polish participants mentioned a feeling of being unfairly treated in Norwegian society and talked about experiencing discrimination. “*I have a lower chance of finding a job or an apartment, and it’s true!” (female, Poland).* Another participant lamented that in relation to the pandemic *“everyone complains that ‘the Polish aren’t getting the vaccine’” (female, Poland).*

Accepting discrimination was somehow linked to historical or cultural reasons. In context of the communist regime, the Polish group explained a mentality of “not rocking the boat” in relations to authorities. *“If your boss says you’ll get a lower wage because you don’t speak Norwegian, we will not protest and say that ‘that is illegal when we have the same qualifications’, we will just say ‘aha’, and move on. Our generation that doesn’t speak Norwegian well enough, we feel a bit worse than other Norwegians. Then we just say ‘yeah, well…’ and move on. We don’t fight back” (male, Poland).*

The Somali described feelings of becoming scapegoats in the media during the pandemic, and felt the news had a destructive way of portraying differences in covid cases in immigrant groups. *“There was more focus on people’s backgrounds than on the spread of disease in itself and measures to reduce that” (female, Somalia). “You miss a lot when you only think about immigrants having higher spread of disease than others. In fact, the pandemic affected the whole world. But many focus on immigrants, and that feels like discrimination” (female, Somalia).*

#### Access to health services

All Polish participants mentioned that it was possible to get the vaccine earlier in Poland than in Norway. This led to many getting vaccinated in Poland. Seasonal workers in Norway do not get a personal ID-number, but instead a substitute D-number. Because of this, many were unable to register for vaccination in Norway. Some of the Polish participants thought this hinderance could explain why some found it easier to get vaccinated in Poland, or not at all. *“The easiest thing was to travel to Poland and get vaccinated there. It is as simple as that*” (*male, Poland).* However, some participants spoke of working with the authorities to reduce barriers to vaccination among fellow immigrants.

To get Polish vaccinations registered in Norway Polish immigrants had to book a doctor’s appointment, which most didn’t feel any need to considering the inconvenience and cost. Language and information barriers were also considered to play a part in many Poles preferring to get vaccinated in Poland. “*To what degree language difficulties and it being hard for them to book an appointment without help might play a part? There might lie something there too” (female, Poland).*

The Sri Lankan group also mentioned that there could be challenges regarding registering online. *“For elderly, some have access to log in online, while some can’t log in online because they don’t know how” (male, Sri Lanka).*

### Other personal factors

None of the participants mentioned factors related to housing, crowdedness or poor health. However, when asked about their thoughts about the spread of COVID-19, the Somali pointed out types of jobs as a factor. *“Most people from Somalia could not work from home. Many are for example bus or taxi drivers, or cleaners, and these services were not stopped” (female, Somalia).*

The Polish group pointed out that many Polish immigrants are labor immigrants who often commute back and forth. During the pandemic, the borders closed, and many could not travel. *“Maybe those who come only to work, they might have seen the whole pandemic as a problem or an obstacle for their work. Not being able to cross the border and such. And they don’t see the risk. Many didn’t care if they had symptoms and might be sick” (female, Poland).* This was mentioned as a possible reason for the spread of the virus.

Two Polish participants also pointed out that education level might play a role in vaccination hesitancy. *“Short time living in Norway and low level of education I think affects the rate” (female, Poland).* Education level was also mentioned in the Sri Lankan group – they believed that since many Sri Lankans have higher education, and are well integrated, it is natural for them to follow advice from the government. *“I think in our group, most people have a job. Either they are students, or they are working. We are active in the Norwegian society and follow rules and regulations” (male, Sri Lanka).*

## Discussion

Our results give a deeper understanding of what immigrants from Somalia, Sri Lanka, and Poland consider ‘cultural factors’ that might be possible causes for a higher COVID-19 disease burden and lower vaccination coverage among immigrants living in Norway. Despite the heterogeneity of responses within the groups, there seemed to be both similarities and differences in what the three groups mentioned as plausible cultural factors, and how they chose to weigh their importance. In addition to cultural factors, like social habits, religious traditions, and societal duty, and transcultural related factors, the immigrants also mentioned structural and systemic factors at the host country level, pertaining to access to services, appropriate information, and discrimination, influencing their behavior in relation to COVID-19.

All groups mentioned social habits as part of their culture related to the pandemic, but the ways of being social was described in various modes. The Somali group particularly weighed social duty towards other Somalis, with a strong motivation for visiting the sick, and with gatherings in homes and other meeting places being important aspects of their lives. Our results give a deeper understanding to previous studies that the way people interact with each other could explain a high disease burden in certain groups. The Somali explained that it took a while before they realized that visiting each other, and visiting the sick could increase the amount of infected people. One anthropological study mentioned that during sickness and death Somalis feel compelled to visit the family of the diseased to be with them and pray, in accordance with Islamic tradition. Social habits are connected to religious practices [20]. This could explain why it took longer before the Somalis changed their social behavior –as there is a strong sense of duty with a strong both religious and cultural foundation that is harder to ‘override’. The Sri Lankan and the Polish group on the other hand had looser social ties and did not describe the same normative social or religious behavior. Differences in social habits between the three groups might be related to the disease burden amongst them – with the Somali group having the highest disease burden [8]. Considering differences in social habits, norms, and religious beliefs, might therefore be key when giving advice about social distancing and other guidelines.

We found a contrast regarding the sense of societal duty, which was more clearly expressed among the Sri Lankan participants. Thus, the term “collective responsibility” used by the government [12] may due to cultural differences, have resonated better in some migrant groups than in others. The Sri Lankan participants all described a strong feeling of solidarity in relation to vaccination. Collectivism in the Sri Lankan culture coincides with a previous anthropological study [21]. This was less articulated amongst the Poles, with participants mentioning that engagement in the broader local community is relatively uncommon. With a more individualistic structure of social relations and community Poles might not have as strong preexisting cultural incentives to follow a vaccine recommendation, when not directly beneficial to themselves.

Adherence to rules and guidelines was described among the Sri Lankans as a cultural factor for them, along with wishing to be viewed as respectable citizens in their community. The Polish participants described a more individual culture, where following government rules and guidelines is not a goal in itself, and as such they may have had a weaker cultural inclination to adhere to guidelines. These attitudes to rule-following echo the tightness levels assigned to Sri Lanka and Poland in a paper examining the relationship between COVID-19-cases and cultural tightness in different countries [22]. Variation regarding cultural tightness, sense of societal duty and attitudes towards rule-following, show how the Sri Lankan and Polish groups may have had differing baseline compatibilities with public health measures.

Natural remedies were mentioned as part of both Sri Lankan and Somali culture. Some may view natural remedies as more important than vaccination for reducing the risk of getting COVID-19 [7], but our participants did not talk about them as in conflict with each other.

A previous study pointed out how degree of integration, knowledge of language, and trust in the system influenced behavior during the COVID-19 pandemic [9]. How quickly one integrates into a new society, how one relates to the system there and the level of trust are all factors that are related to both the culture of the country of origin and the destination. Distrust in public health authorities has been described as a barrier towards vaccination that was amplified during the COVID-19 pandemic [11, 16]. Polish immigrants in Norway explained that they as a group tend to have low trust in the healthcare system, consistent with the findings of others [23]. After the fall of the communist regime, Polish people became more secluded around their tight circles of family, trusting more in their family and friends than in social political structures [24]. The Sri Lankan participants reported a high level of trust in the healthcare system. This discrepancy in trust levels aligns with the differing rates of COVID-19 vaccine uptake among Poles and Sri Lankans, with Poles demonstrating lower rates and Sri Lankans higher rates. However, trust is a dynamic virtue, and the evolvement of trust during the pandemic has also been described [14]. However, this topic was not touched upon in our material.

Lack of language proficiency was identified by participants as a key barrier to adherence to guidelines. Polish and Somali populations would have a harder time navigating health information than Sri Lankans, who had a good fluency. Language is also linked to education and health literacy [25]. It has previously been pointed out that cultural differences in regard to exchange of information could lead to barriers to obtaining information [8]. Lack of appropriate information was also pointed out as a factor that impacted the Somali participants negatively during the COVID-19 pandemic. The transcultural aspect of being connected both to the culture at home and in a new country may be a source of confusion or doubt, as brought up by the Polish participants in our study. However, it may also be the most relevant source of information in the lack of accessible and understandable information in the host country. Understanding how information is obtained and distributed within immigrant groups, and further facilitating for learning the language, might reduce tensions and uncertainty.

A challenge for immigrants, is that policymakers are often not familiar with their cultures and different ways of life. Successful integration might make it easier for immigrants to voice concerns about how guidelines might affect their quality of life negatively in ways not obvious to policymakers [26, 27]. Also, there must be apparent advantages to putting in the oftentimes vulnerable and demanding effort to integrate in a new society. Therefore, the continued effort to reduce barriers on the side of society to immigrants’ integration remains important.

Type of work was considered by the Somali to be an important reason for forced virus exposure risk. In addition, most Somali immigrants in Norway came as refugees with a low education level [28]. Such a disadvantaged socioeconomic background, along with the previously discussed language barriers, has been mentioned in the literature as an explanation for lower health literacy, leading to a higher pandemic burden of disease [8, 25].

Both Somali and Polish participants spoke of feelings of their nationalities becoming scapegoats in media and public discourse with regards to spread of disease and vaccination, respectively. In a previous qualitative study, Somali participants pointed out that negative focus in the media, based on immigrant background in relation to COVID-19, could lead to stigma and fear of getting sick or admitting to being sick [8], and this could weaken the sense of societal belonging previously described as a factor influencing guidelines adherence.

Although immigrants’ cultural background may shape their response to national COVID-19 guidelines, the immigrant reality characterized by degree of integration and being ‘between cultures’ is of great importance. The Sri Lankans’ desire to successfully integrate into Norwegian society, greatly influenced by cultural values, is an example of such an interaction. A multidisciplinary approach making use of social anthropology would seem useful in future research to better understand what lies in the term cultural factors.

Structural barriers to vaccination for immigrant groups have been pointed out in previous literature [14]. Our results indicate structural barriers for the Polish group, which imply that the official vaccination rate for that group may be lower than the actual rate.

Strengths of this study are a somewhat large number of participants, with relatively varied demographics, and consulting three of the larger immigrant populations in Norway. Although this allows us some generalizations of results according to the three countries of origin, we are aware of the heterogeneity within groups and all our results should be interpreted with caution not to stigmatize particular groups or subjects. Furthermore, in terms of weaknesses, we had a narrow age range among participants, the gender distribution was different in the three groups and the duration of immigration varied, despites our efforts to have a broad spectrum in regard to these characteristics. These efforts also made us choose a combination of individual and group interviews. Groups offered an opportunity to get people to be inspired by others, while the individual interviews provide more privacy to present possible different ideas. However, qualitative studies do not intend to be representative of the populations but rather gather different experiences and opinions of the groups under study. Larger, or different groups of participants could have changed some of our results.

An obvious possible barrier when interviewing migrants is language. To mitigate this, we offered the possibility of having a translator present. In one interview, we used an official interpreter. This could influence the way participants answered, and the perceived meaning of their answers. Finally, our findings are not generalizable for the different immigrant groups or for immigrants in general.

## Conclusion

Understanding how migrants interpret and consider cultural factors in relation to the pandemic is crucial to increase trust and compliance in times of health crises. Rules and guidelines may hit harder and interfere more in the way of life in some communities than others. In the continued work towards equity in health promotion and healthcare services, policymakers ought to keep the existence of such cultural differences in mind, to be able to make policies well fitted to ensure good health and quality of life for all.

### Electronic supplementary material

Below is the link to the electronic supplementary material.

Supplementary Material 1

## Data Availability

The data generated from our research is not publicly available, but anonymous transcripts may be shared upon specific request and approval from the Norwegian Agency for Shared Services in Education and Research.
