# Supplementary material for: Cultural factors influencing COVID-19-related perceptions and behavior, seen from immigrants’ own perspective – a qualitative study in Norway
Source: Arch Public Health. 2024 Jul 19;82:110. doi: 10.1186/s13690-024-01327-z (PMC11264612; doi:10.1186/s13690-024-01327-z)
Supplement: Supplementary file 1 — Supplementary Material 1 [file 13690_2024_1327_MOESM1_ESM.docx]

**Interview guide – English translation**

Introduction: Thank you for participating! Confidentiality and consent form.

1. Introduction: Reports from the Norwegian institute of public health show there has been a relatively high fraction of immigrants getting COVID-19 disease, compared to the rest of the population. It has also been observed that certain immigrant groups have a lower vaccination coverage than Norwegian-born with Norwegian parents. Studies that have been done, and reports from the Norwegian institute of public health point to cultural differences as one possible explanation for differences in incidence of COVID-19 disease and vaccination rate between immigrants and the rest of the population, without exploring what such factors might entail. We wish to examine what you participants think about this, to get a better understanding of the relationship between culture and health, because we want a health service that is more accessible to everyone.

In this setting we define immigrants as persons with permanent residency in Norway, but who are not born here.

1. Background questions for all participants
   1. Country of origin?
   2. Time lived in Norway?
   3. Age?
   4. Living conditions (alone/with family)?
   5. Area of work/studies?

**Questions for Somali group:**

- In the first phase of the pandemic it was observed seven times as many confirmed cases of infection per 100 000 among Somali immigrants, compared to Norwegian-born without immigrant backgrounds.
  - What do you think about this difference?
  - What cultural factors do you think might explain that Somali immigrants were overrepresented among infected?
    - (for example shame/stigma?)
    - (language/integration)
    - (discrimination)
  - A study looking at corona infections in Oslo’s districts mentioned that «it might be that cultural/ethnical background in itself is a factor in the spread of infection. For example, there may be other norms for physical or social closeness, or there may exist systematic misconceptions about infection and measures to prevent infection» - What do you think of this quote?
  - Are there any things you think researchers are overlooking when they examine why there has been high infection rates among immigrants from Somalia?
  - Have you noticed any measures to prevent infection that have been targeted to immigrants from Somalia?
    - If yes, how do you feel they have worked?
  - What measures do you yourselves think might have helped to reduce infection rates?
  - Lastly – do you have any other thoughts on how culture might affect health in a pandemic?

**Questions for Polish group:**

- In October 2021 it was found that the vaccination rate among Polish immigrants was approximately 43%, compared to 90% among Norwegian-born without immigrant backgrounds.
  - What do you think about this difference?
  - Are there any things you think might explain the low vaccination rate?
  - In a study examining vaccination coverage among immigrant groups it was mentioned that «there may be underlying factors like integration and time of residency that affect willingness to get vaccinated» - What do you think about this quote?
  - Are there any things you think researchers overlook when trying to examine why there was a lower vaccination coverage among immigrants from Poland?
  - What cultural factors might explain the vaccination rate of immigrants from Poland?
  - Have you noticed any measures to increase vaccination coverage, that have been specifically targeted to immigrants from Poland?
    - If yes, how do you feel they have worked?
  - How do you experience that immigrants from Poland deal with Norwegian health services (general practitioners/hospital etc.)?
  - What measures do you yourselves think might have helped to increase the vaccination coverage?
  - Lastly – do you have any other thoughts on how culture might affect health in a pandemic?

**Questions for Sri Lankan group:**

- In October 2021 it was found that the vaccination rate among Sri Lankan immigrants was approximately 91%, compared to 90% among Norwegian-born without immigrant backgrounds.
  - What do you think about this high vaccination rate?
  - Are there any things you think might explain the high vaccination rate?
  - In a study examining vaccination coverage among immigrant groups it was mentioned that «there may be underlying factors like integration and time of residency that affect willingness to get vaccinated» - What do you think about this quote?
  - What cultural factors might explain there being a high vaccination rate among immigrants from Sri Lanka?
  - Have you noticed any measures to increase vaccination coverage, that have been specifically targeted to immigrants from Sri Lanka?
    - If yes, how do you feel they have worked?
  - How do you experience that immigrants from Sri Lanka deal with Norwegian health services (general practitioners/hospital etc.)?
  - What measures do you yourselves think might have helped to increase the vaccination coverage?
  - Lastly – do you have any other thoughts on how culture might affect health in a pandemic?
